# Women's interpretation of and responses to potential gynaecological cancer symptoms: a qualitative interview study

**DOI:** 10.1136/bmjopen-2015-008082

**Published:** 2015-07-06

**Authors:** E L Low, K L Whitaker, A E Simon, M Sekhon, J Waller

**Affiliations:** 1 Department of Epidemiology and Public Health, Health Behaviour Research Centre, University College London (UCL), London, UK; 2 School of Health Sciences, University of Surrey, Guildford, Surrey, UK; 3 Centre for Health Services Research, City University, London, UK

**Keywords:** PREVENTIVE MEDICINE, PRIMARY CARE, QUALITATIVE RESEARCH, PUBLIC HEALTH, GYNAECOLOGY

## Abstract

**Objective:**

To explore women's experiences of symptoms potentially indicative of gynaecological cancer in a community-based sample without imposing a cancer perspective.

**Design:**

A qualitative interview study with thematic analysis of transcripts.

**Participants:**

26 women aged ≥30 years, who had experienced a symptom that might indicate gynaecological cancer in the past 3 months, were recruited using a screening questionnaire distributed online and in community settings.

**Setting:**

London, UK.

**Results:**

Women attributed gynaecological symptoms to existing illnesses/conditions or considered themselves to be predisposed to them, either through their ‘genes’ or previous personal experience. Normalising symptoms by attributing them to demographic characteristics (eg, age, sex) was common, as was considering them a side effect of hormonal contraception. When women raised cancer as a possible cause, they often dismissed it as unlikely. Responses to symptoms included self-management (eg, self-medicating, making lifestyle changes), adopting a ‘lay system of care’, or consulting a healthcare professional. Triggers to help-seeking included persistent, painful or debilitating symptoms, concern about symptom seriousness, and feeling that help-seeking was legitimised. Barriers to help-seeking included lack of concern, vague symptoms, unusual symptom location, competing time demands, previous negative experiences with the healthcare system, and not wanting to be perceived as a time-waster.

**Conclusions:**

Attributions of symptoms potentially indicative of a gynaecological cancer were varied, but most often involved women fitting symptoms into their expectations of what was ‘normal’. Normalising acted as a barrier to seeking help from a healthcare professional, alongside competing time demands and negative attitudes towards help-seeking. These barriers may lead to later diagnosis and poorer cancer survival. Our findings could be used to inform the development of interventions to encourage appropriate help-seeking.

**Strengths and limitations of this study**

- This is the first qualitative, community-based study to assess how women interpret and respond to symptoms possibly indicative of a gynaecological cancer outside the cancer context.
- The model of pathways of treatment was used to frame the interview schedule and interpret the findings to allow for comparison with other research.
- Women often interpreted their symptoms as ‘normal’, attributing them to their gender, age or to the use of hormonal contraception. Responses to symptoms included self-management, adopting a lay system of care or consulting a healthcare professional.
- The sample was homogenous; most of the women were white and from relatively high socioeconomic backgrounds.
- Larger and more demographically diverse studies are needed to clarify how women interpret and respond to symptoms possibly indicative of gynaecological cancer.

## Introduction

Gynaecological cancers have a combined annual incidence second only to breast cancer in UK women.^1–4^ There is increasing evidence that earlier diagnosis of gynaecological cancers could contribute to improving the survival gap between the UK and other countries with similar healthcare systems.^5^ ^6^ Routes to earlier diagnosis of gynaecological cancers are currently limited, with screening only available for cervical, but not other forms of gynaecological cancer.^7^ Therefore, one avenue for earlier diagnosis is encouraging prompt help-seeking.^8^ Retrospective evidence from patients with gynaecological cancer suggests that symptoms that are ‘alarming’ such as bleeding or pain trigger help-seeking, while non-recognition of symptom seriousness, misattribution of symptoms to non-serious or benign causes, lack of awareness, fear, and worry about wasting the doctor's time act as barriers.^9–13^ In one of these studies, a ‘triggering process’ was described where the normality of novel bodily sensations was challenged, transforming them into symptoms in need of care. Important elements in this process included normalising, level of severity and interference, competing social responsibilities and social legitimisation.^11^

However, retrospective evidence from women with a cancer diagnosis, while important, may not wholly reflect how people respond to symptoms when they first occur. Community-based studies have found that although intention to seek help is high in women who anticipate having a symptom of a gynaecological cancer,^14^ ^15^ actual help-seeking is lower in women with the same symptoms when they are asked about it without mentioning cancer.^16^ Findings from this population-based survey also showed that there were potentially many more women in the population with possible gynaecological cancer symptoms than those who are seeking help for these symptoms.

To date, in-depth research to explore how women interpret and respond to bodily changes—as they are experienced—has been limited. A previous focus group study explored some of these issues in women and found the most common reason for foregoing medical help-seeking was the belief the symptom was benign.^17^ However, anticipated and actual help-seeking were not always differentiated, and the study was conducted in the US, which has a different healthcare system compared with the UK.

We used the model of pathways to treatment (MPT) as a theoretical framework in the present study, which identifies contributing factors that may influence a patient's pathway to treatment. The model describes events, processes and intervals; from detecting bodily changes (appraisal), perceiving a reason to contact a healthcare professional (HCP; help-seeking), through to the first consultation, diagnosis and the start of treatment.^18^ The present study was novel in its exploration of recently experienced gynaecological symptoms reported by women in the community, discussed without imposing a ‘cancer’ frame.

## Methods

### Participant selection and recruitment

We recruited participants in London through an online screening questionnaire disseminated to women via non-medical settings during 2012. Online settings included the Mumsnet and Streetlife websites. In addition, posters displaying the web address of the screening questionnaire were displayed in a range of locations, including job centres, libraries and community centres. We aimed to recruit a varied sample of women of different ages and from different socioeconomic backgrounds and ethnic groups. Potential participants were told that the study was about women's health and were invited to complete the screening questionnaire.

The questionnaire asked about women's experiences of 14 gynaecological symptoms identified as possibly indicative of cancer through National Health Service and cancer charity websites (see table 1) in the past 3 months. Women also provided demographic information. At the end of the questionnaire, women were asked whether they would be willing to be contacted about taking part in an interview to explore their health experiences in more depth. Interested women provided their contact details.

**Table 1:** Demographic characteristics and symptom reporting of women interviewed (n=26)

	n	Per cent*
Age group (years)		
30–39	9	35
40–49	6	23
50–59	5	19
60–69	6	23
Ethnicity		
White British	22	85
White other	4	15
Non-white	0	0
Education level		
Degree or higher degree	16	61
Higher education qualification below degree level	2	8
A levels or highers	2	8
ONC/BTEC	2	8
O level or GCSE equivalent	3	11
No formal qualifications	0	0
Other	1	4
Car ownership		
None	7	27
One or more	19	73
Home ownership		
Own outright	7	27
Own with mortgage	12	46
Rent from local authority/housing association	2	8
Rent privately	4	15
Other (eg, living with family/friends/ squatting)	1	4
Symptoms reported		
Pain in abdomen/lower back/pelvis	14	54
Increased abdominal size	12	46
Increased need to empty bladder more often/urgently	13	50
Increased wind or constipation	12	46
Difficulty eating/feeling full quickly	6	23
Heavier/longer periods	11	42
Changes in bowel habit	10	38
Pain/discomfort during sex	7	27
Itching, pain or soreness of vulva	7	27
Bleeding between periods	6	23
Discharge that smells unpleasant or is blood stained	7	27
Bleeding during/after sex	6	23
Growth/lump/sore/ulcer on skin of vulva	7	27
Bleeding after menopause	0	0

*Most participants reported more than one symptom so the total % for symptoms is >100%.

BTEC, Business and Technology Education Council; GCSE, General Certificate of Secondary Education; ONC, Ordinary National Certificate.

A total of 123 women responded to the screening questionnaire, of whom 70 reported at least one eligible symptom in the last 3 months, and agreed to an interview. From this pool, purposive sampling was used to select women from a range of age, ethnic and socioeconomic status (SES) backgrounds. Most women who responded were, in fact, white, educated to mid-level or high-level, and from high-SES groups (indexed by home and car ownership). In total, 26 were interviewed (37% of those identified as eligible). We had originally planned to interview 20 high-SES and 20 low-SES women, but were unable to recruit more women from low-SES groups, and chose not to interview more women from similar backgrounds to the higher SES participants as we had reached data saturation within that group.

At the end of the interview women were encouraged to contact their doctor if they had any persistent symptoms.

### Interviews

ELL conducted the interviews. Participants chose either a face-to-face interview at University College London (UCL) (n=10), a neutral location where they could discuss their (potentially sensitive) symptoms, or a phone interview (n=16) if they felt uncomfortable either speaking about their symptoms face-to-face or were unable or unwilling to travel to UCL. All travel expenses were reimbursed.

The average duration of the interviews was 35 min. A semistructured topic guide, underpinned by the MPT, was used to explore themes related to symptom appraisal and help-seeking. Women were probed about what they thought caused the symptom (with no mention of cancer by the interviewer), and what action they took. The interviews were digitally recorded and were transcribed verbatim by a professional freelance transcription service. Transcripts were checked against portions of each digital recording for accuracy. Once transcription was finished, ELL read and re-read the transcripts to check the integrity of the data.

### Analysis

Transcripts were analysed thematically using guidelines outlined in Braun and Clarke,^19^ within the NVivo software package. ELL and MS read and re-read the transcripts and generated initial codes. These were discussed in frequent meetings with ELL and JW. Themes were further categorised into the appraisal and help-seeking intervals defined in the MPT, with agreement from all coauthors. The coding framework was refined a total of eight times in an iterative process. Within the final framework, two broad themes were identified, each with a number of subthemes (table 2).

**Table 2:** Thematic structure mapped on to appraisal and help-seeking intervals of the model of pathways to treatment^18^

Interpretation of symptoms (appraisal interval)	Response to symptoms (help-seeking interval)
*Patient factors* Normalising	*Patient factors* Self-management Adopting a lay system of care Competing demands
*Disease factors* Existing illnesses/predisposed Cancer as a possible cause	*Disease factors* Perceived seriousness Persistence Previous symptom experience
	*Healthcare provider and system factors* Worry about wasting the general practitioners' time Difficulty in getting an appointment Gender of the general practitioner

## Results

### Sample characteristics

Demographic characteristics of the sample are shown in table 1. A broad range of age-groups were represented, with 35% in the 30–39 year age group, 23% in the 40–49 year age group, 19% in the 50–59 year age group and 23% in the 60–69 year age group. The majority of women were White British (85%) and most were educated to degree level or higher (61%). The most common symptoms reported by women were pain in abdomen, lower back or pelvis (reported by 56% of women) and an increased need to empty bladder (50%). The majority of women reported more than one symptom (mean=3, range=1–8).

### Interpretation of gynaecological symptoms

#### Patient factors

##### Normalising

Women often appraised their bodily changes as normal, and simply a consequence of diet, being female or *down to age* (OL02, bleeding after sex, age 50 years).

That's probably, again, linked to my periods because it's worse at certain times of the month … I've just always thought that maybe it's, kind of, diet and, kind of, linked with my periods. A lot of my friends … complain a bit as well so I just, kind of, think that it's something that's fairly common … It was just, kind of, one of those things that you just think, well, that's part of being a woman, really. (OL02, abdominal bloating, age 46)

I’m right in the process of kind of menopausing … periods are getting less and less and less and less. So I think they’re sort of departing. They may even have gone by now, I may actually have had the last one. So it's a very … it’s actually really difficult to discern kind of what’s bleeding and what’s period residue. (OL01, bleeding after sex, age 50)

Other normalising explanations included using hormonal contraception. In these cases a number of women had already been warned that their symptom may be a side effect of the medication they were taking. Therefore, when they did experience that symptom, it was logical for them to attribute it to the medication.

I have a contraceptive implant which can cause irregular bleeding and I have had it since April. It never caused me any trouble and suddenly all this. So that could be one of the reasons. That would be the obvious reason. (OL37, heavier or longer periods than normal, age 30)

#### Disease factors

##### Existing illnesses/predisposed

Women often attributed their symptom to an existing or past condition, illness, disease, surgery or injury. These attributions suggest that women will attempt to ‘fit’ new symptoms to existing illness schemas, at least in the first instance.

I probably blame the fibroids … These things are, kind of, crowding me out, I can't eat, I can't hold my urine, I can't do anything, I'm heavy, I'm bloated. So I guess the fibroids are what I would, kind of, blame logically first. (OL04, heavier or longer periods than normal, increased abdominal size, discomfort in the abdomen, increased need to empty bladder more often or urgently and difficulty eating, age 46)

One woman referred to her previous experience of bleeding easily when interpreting bleeding during sex.

I think skin sensitivity, I honestly do. I feel that this is minor bleeding on the inside of the vagina. I … have excessively sensitive skin on the outside and also in my nose and I know that sounds weird but it seems to me that if you’re kind of sensitive on the outside, why might you not be on the inside? You know, if I blow my nose here, it will bleed. I mean, I often get blood there. Not nose bleeds, but just blood. It’s, so going back to vaginal bleeding, you can see why it’s not something that would disturb me unduly. (OL01, bleeding during sex, age 50)

Women described feeling predisposed to experiencing symptoms and this was related to their family history. “I think it’s hereditary, you see, because my mother had this problem as well” (OL01, increased need to empty bladder, age 50).

Just like my mum and my auntie, we are all a bit of a likeness that way … because members of your family have experienced the same sort of problem as they have got older and it hasn't meant anything, you know, there's been no problem associated with it, you think, oh I'm just getting older and it's a family thing and I don't worry about it. (OL33, increased wind, age 60)

Stories of the influence of family history were sometimes quite elaborate, and were not always based on a previous diagnosis or condition. For example, one woman who reported pelvic pain around the time of her period disclosed that dizygotic twins ran in her family. In this context, she believed that the pain was caused by the release of two eggs during ovulation. This attribution was then further cemented by information from her grandmother.

My nan reckons that eggs are released from both sides or a double egg comes from one side every other month … She says that's why twins run in the family … everybody was really surprised when I was pregnant that I … didn't have twins because … I had got those pains … That's why I have been told that I get those pains, because I get a bad pain on my left-hand side every other month. (OL26, pelvic pain, age 34)

##### Cancer as a possible cause

When cancer was raised as a possible cause, the attribution often formed part of a cycle of possible attributions and was dismissed as unlikely. Women doubted their cancer attributions, concerned that they were over-reacting.

I don't know, if I have stomach cancer or, I don't know, anything like that, because everything seemed to be getting better and I, kind of, thought it was probably to do with the uterus and the fibroids. Whether I should have gone and spoken to the doctor about things as well? I don't know. I don't like going to the doctor more than I need to. (OL04, increased abdominal size, discomfort in the abdomen, age 46)

I am either going through the menopause or I have got cervical cancer. And then, for some reason, I decided that I could possibly have chlamydia, not that that's got anything to do with your periods or the fact that I was at risk of getting it but I'm just, erm, a bit of a worrywart, really. (OL16, heavier or longer periods than normal, age 41)

The unusual location of reported lumps also led women to dismiss cancer as a possible cause, as it did not ring ‘alarm bells’.

I wasn't particularly concerned about it … if I find a lump in my breast, then alarm bells start ringing and you start going, oh my gosh, I've got to see the doctor straight away … it maybe fleetingly went through my mind that it would be something that … needed investigation, yeah, a bad lump, a cancerous lump or something like that … it's not causing any problems … it hadn't made alarm bells ring in my mind so I just want to see what happens to it, rather than rushing to the doctor and freaking out. (OL06, lump on vulva, 34)

### Responses to gynaecological symptoms

#### Patient factors

##### Self-management

Self-management was a common response to symptoms and an alternative to seeking medical help, although the reasons or justifications women gave for these decisions varied. The language used by some women suggested that they viewed their symptoms with a ‘stiff upper lip’, deciding that they should not let these interfere with their lives, even if these were causing discomfort or concern.

I would say in my mid-40s it started to bother me. I mean, it is a bother but, you know, you just put up with it, don't you? (OL21, increased wind, age 62)

Other women described self-management as a way of ruling things out, or deciding whether medical attention was needed.

I took some motilium and, kind of, moved on with my life and, sort of, figured if it went on for any longer then I probably would go and see my GP because that would be worrying. (OL07, increased abdominal size that does not go away (including bloating), age 33)

Some of these women had an idea about what might have caused their symptoms, which appeared to influence their response to it.

I think I'd probably try and sort myself out first with eating and say, right, okay, that's enough of dairy … and then I'd see how it went from there and then if I thought I needed to go to my GP, I'd head off there. (OL27, increased abdominal size, age 52)

For a few women, their decision to put up with their symptom or ignore it seemed to be related to the impact it had on their lives.

It's not frequent enough, it doesn't give me any trouble, so, as I say, I'm inclined just to ignore it. (OL33, increased wind, age 60)

However, even symptoms that were considered quite interfering would be self-managed, with some women setting high ‘tipping-points’ for when they would consider it time to contact a healthcare professional:

Once the pain has gone, you just tend to think, well, okay, there's not really any point. If I get it again, the third time will definitely be the cut-off and I will go and see if they can refer me on. (OL02, lower back pain, age 46)

It would have to go on for a lot longer and be a lot more urgent … And if I was wetting myself or, you know, partially wetting myself then I would be conscious of smelling or things like that. I wouldn't, sort of, suffer in silence but perhaps having to get up every hour in the night or … if it's twice a night then I can put up with it. (OL10, increased need to urinate more frequently and urgently, age 39)

One of the most common non-medical self-management techniques that women mentioned was to manage their symptoms with food or drink remedies, including avoiding certain foods or drinks or introducing others into their diets. Women who reported using these self-management techniques mostly did so in response to changes in their bowel habits or a persistently increased abdominal size, including bloating. This suggests that women believed that their symptoms were related to their digestive system, and that these could be resolved by altering their eating or drinking habits.

I don't take laxatives or anything like that. I try to do it through what I eat, through roughage. I don't know whether it's a lazy gut or whatever because sometimes it can be violent and other times. (OL43, changes in bowel habit, including constipation, age 64)

In order to really make sure that I’ve eaten lots of good solid food that’s going to keep me strong and keep me moving around, cos I walk and … I do a lot of stuff. I really do rely on fairly kind of high fat stuff. (OL01, difficulty eating, age 50)

##### Adopting a lay system of care

Women talked about seeking help via the ‘lay system of care’^20^ which included family, friends and others in their social network. Some women described how the experiences of others influenced their appraisals and subsequent decision to seek help. For example, one woman suspected that her irregular bleeding was caused by her contraceptive implant, and described seeking advice from a friend who had the same contraceptive device fitted.

I spoke to my friend who had it as well and she said, “Oh, just take [the contraceptive implant] out, it will stop.” So it has, hopefully. (OL37, heavier or longer periods than normal, age 30)

Although the ‘lay system of care’ describes help-seeking from various social connections, women appeared to be particularly influenced by close family members. Their advice perhaps gave more depth to the potential consequences of a bodily change or symptom compared with non-relatives possibly because of shared genes. For example, one woman talked about the importance of seeking advice from close relatives so as to gauge the level of health threat:

I talked to my mum and my sister about it and my mum said, oh yeah, it’s cystitis, cystitis. And I said, it’s not … I think it’s hereditary … I just tend to think that it’s always worth asking your mother and your sister about these things because a lot of these things are quite similar within families and if they appear to have the same thing and they’re still alive, why should I not be? (OL01, increased need to empty bladder, age 50)

##### Competing demands

Other common reasons for avoiding seeking help included not prioritising help-seeking or putting it off. Competing demands included other more immediate healthcare needs or wanting to put others first. One woman talked about having several other ailments that required her attention and prevented her from seeking help for vaginal bleeding:

I have the procession of minor ailments permanently, that are really annoying and sometimes really quite debilitating … it’s all these things that there’s always something more immediate … that’s what stops me. It’s not to do with not wanting to tell the GP … it keeps moving down the list of priorities cos something else takes precedence. (OL01, vaginal bleeding after sex, age 50)

As well as other ailments acting as competing priorities, people discussed putting other people first, which outweighed seeking help for their own bodily changes.

My husband is being investigated for prostate cancer … he comes first for something like this. His needs would be beyond mine … in terms of supporting him … that would be a barrier … his needs along with the needs of my children would come first. (OL14, increased wind, increased abdominal size, changes in bowel habit, age 46)

#### Disease factors

Disease-related factors that influenced help-seeking were perceived seriousness, persistence and previous symptom experience.

##### Perceived seriousness

If a symptom was perceived to be getting worse, this was sometimes a trigger to seeking medical help.

I basically went back recently because of how bad it had got … I would say over the last year it was probably getting slightly worse each time … Tiredness, and headaches I was getting as well, so, sort of, just generally feeling really out of sorts for a good few days each month and beforehand, sort of, mood swings … and I think they [periods] were getting heavier, or it felt like they were. (OL03, heavier or longer periods than normal, age 34)

Social comparison could also serve to reinforce the perceived seriousness of symptoms and legitimise help-seeking:

I … saw other people's experiences and that … persuaded me that mine was bad enough to actually do something about it and that I wasn't just making a fuss about nothing … I, sort of, realised yes, it probably is quite bad … that was probably a factor in doing something about it as well. (OL03, heavier or longer periods than normal, age 34)

Awareness that a symptom could be indicative of cancer was also a trigger for help-seeking:

I had read that if you get bloating and it doesn't go away, that is usually a sign maybe that's cancer. If it went down, which happens to me, it probably isn't … There is quite a history of cancer in my family … I was frightened so it prompted me … I think I am probably like a lot of people who think, oh it will go away, it will be fine, don't worry, but it didn't and … I got scared. (OL43, persistent bloating, age 64)

Conversely, for those not perceiving their symptom as serious, they did not perceive value in contacting a healthcare professional because they felt that what they were experiencing was just something that just ‘happens’:

I guess I have not thought enough to be able to actually go and get it sorted out or, you know, speak to anybody about it, I just, kind of, assumed, you know, that might be what happens. (OL24, bleeding between periods, age 35)

##### Persistence

Symptoms that were not persistent or long-lasting prevented women from help-seeking, and this was linked to concern about bothering the general practitioner (GP) unnecessarily.

I think it would have to be more persistent and last for longer for me to think it was worthwhile to bother the GP. You always feel apologetic when you see a GP. (OL31, constipation, age 62)

##### Previous symptom experience

A final reason for putting off seeking medical attention was comparing the symptom with previous experiences. For example, one woman had previously experienced severe pancreatitis. This previous experience of a painful and acute illness influenced her judgement of later symptoms.

I have been with the pancreatitis, that was drastic … obviously, that's immediate. Whereas this isn't quite immediate and I think, actually, that's a bad thing for me because I tend to judge everything by that previous experience, say, well if it's not like that, it's not as urgent, it's not as important, which isn't a good thing … I have been blowing it off a bit. (OL14, heavier or longer periods than normal, age 40)

Previous negative test results could also lead women to feel that their symptom was not important, and prevent help-seeking:

Given that, you know, I have had so many tests that were negative in the past, it's again this issue — am I going to bother the GP with something that's going to come up negative again? So I probably wouldn't go. (OL31, discharge that smells unpleasant, age 62)

#### Healthcare provider and system factors

Women reported visiting several types of HCP, including GPs, pharmacists, genitourinary medicine clinics or accident and emergency departments. A number of barriers to seeking medical help were raised, and are described below.

##### Difficulty making an appointment

For this woman, making an appointment was so difficult and time with the GP so short that she had not sought help for her symptom:

It would have to be easier to get an appointment with the GP. It really is that, that is such a bloody drama. And you get you know, thirty seconds with your GP and you’re allowed to talk about one thing. (OL01, vaginal bleeding after sex, age 50)

##### Wasting the doctor's time

Another barrier that women mentioned was a concern about wasting GP time. For most of these women, this stemmed from a belief that their symptoms were not serious enough or there was inadequate justification to “bother the doctor” (OL36, increased wind, age 53).

I would do. I'm sensible. I'm not going to be stupid about it, but, on the other hand, I don't want to bother people because there are people who are really ill. (OL28, itching, pain or soreness of vulva, age 57)

##### Gender of the GP

Finally, for a number of women in this study, the gender of their GP was mentioned as a contributing factor:

It's important because I think you need to be able to say … like you can sit down and explain things when they are sometimes quite embarrassing, to this person. (OL30, lump on vulva, age 32)

Other women discussed visiting a male GP as a last resort because they would feel embarrassed about talking to a man about their symptoms:

I think it would have to become really bad. Because he's the male GP as well and I would feel a bit embarrassed talking to him in a way. (OL36, increased wind, age 53)

## Discussion

This is the first qualitative study to explore how British women appraise and respond to changes in their bodies that may indicate gynaecological cancer, outside the cancer context. Attributions of bodily changes were often aligned to women's expectations, either based on previous illness experience, perceived hereditary conditions or their expectations of getting older or being a woman. These findings are consistent with evidence from interviews with patients with cervical cancer^10^ and support previous research in patients with melanoma, where prior beliefs and experience were important determinants of help-seeking. For example, if a change in a mole did not meet the patient’s expectations of a ‘bad’ sign (eg, bleeding, itchy mole), the appraisal interval was prolonged (ie, time from noticing a bodily change to deciding to consult a healthcare professional).^21^

For bodily sensations that were not consistent with expectations, women sometimes mentioned cancer as a possible cause, but it often formed a cycle of changing attributions and was usually dismissed by women as unlikely. For others, unexpected symptoms were often not attributed to anything specific, and were dismissed as trivial and not worthy of further consideration, mirroring findings from studies with patients with cancer.^9^ ^10^ ^12^ Symptom attribution was associated with women's responses to their symptoms. If symptoms were considered ‘normal’, the typical response was to self-manage. Women also relied on their lay networks for support and guidance in respect of their symptoms, particularly from close family members, which has long been recognised as a trigger to consultation.^22^ A similar theme was recently observed in a qualitative study with patients with cervical cancer in Uganda; conversations with husbands, relatives and friends reinforced women's determination to seek medical help.^23^ The importance of social networks for prompting help-seeking has previously been highlighted,^24^ and may be a promising avenue for intervention work aimed at encouraging help-seeking across sociodemographic groups.^25^

Consulting a healthcare professional was discussed in terms of triggers and barriers. Symptom severity and persistence triggered help-seeking from a healthcare professional, while non-recognition of seriousness and previous symptom experience could undermine it. These findings echo those from studies of people with a cancer diagnosis, particularly the intermittent/vague nature of symptoms.^26^ They also support a recent review of the literature highlighting the possible impact of previous symptom experiences on subsequent help-seeking.^27^ System barriers were also similar to those found in previous research, including difficulty making appointments and worry about wasting the doctor's time.^28^

There was evidence of high tolerance of symptoms which has also been observed in previous studies.^28^ Women reported setting themselves a ‘tipping point’ of when a bodily sensation would trigger help-seeking and this could be extreme, for example, waking up every hour through the night to go to the toilet.

Women also described the role of competing demands, which led to avoidance of help-seeking. These varied from having other more urgent health needs or prioritising the health needs of others. Competing family and work demands have also been given as reasons by women for not seeking help for breast cancer symptoms.^29^ ^30^ One possibility is that women may ‘contain’ or sideline bodily changes to prevent them from interfering with normal life.^31^

### Strengths

One of the main strengths of this study lies in the exploration of symptom appraisal and help-seeking outside of the context of cancer and in non-medical settings. This is also the first time symptom research with a community sample has focused on a specific group of cancers. The exploration of these processes in this way may have led to these findings that more closely reflect real-life responses than research in which women are aware that the symptoms being explored may indicate cancer. The present study explored help-seeking for symptoms potentially indicative of all five gynaecological cancers, which provides a basis for future, larger, quantitative research in this area.

There is very little research in this area which has drawn on a theoretical model to guide the research design and interpretation of the findings.^32^ The current study drew on the MPT, which allowed us to map our findings in a structured way, which in turn, will allow for comparison with future research, and aid the development of future interventions for targeting specific barriers to earlier presentation.

### Limitations

Most of the women in our sample were white, educated and came from relatively high-SES backgrounds and therefore, we cannot rule out the possibility that different themes would have emerged had we been able to recruit a sample that was more diverse with respect to SES and ethnicity. However, we did reach data saturation within our relatively homogenous sample.

### Implications

The findings from the present study help to progress our understanding of how women interpret and respond to symptoms that may be indicative of a gynaecological cancer. However, there is evidence that some of these symptoms can be common,^16^ ^33^ and most are likely to be indicative of benign disease rather than cancer;^34^ so women, mainly, were correctly attributing their bodily changes to benign conditions. Encouraging all women with these symptoms to seek help because of a potential risk of cancer may lead to unnecessary levels of fear. Evidence suggests that GPs have concerns over encouraging help-seeking among all women with symptoms that may potentially indicate a gynaecological cancer due to associated increased costs and demand on resources, as well as the emotional impact on the patient.^35^ Future research might explore how GPs can encourage appropriate help-seeking in those women at higher risk, in particular by challenging ‘normalising’ behaviour. However, help-seeking for symptoms that do indicate a benign condition may be beneficial in its own right, by providing women with reassurance or treatment for other conditions.

### Conclusion

The current research demonstrates that women will adopt alternative strategies to cope with symptoms that are potentially indicative of a gynaecological cancer. Among the most common response was to use self-management techniques and to adopt a lay system of care. The main triggers for contacting a HCP were severity and persistence, which also related to social sanctioning by people in women's networks. Understanding the barriers and triggers to help-seeking at a cancer-specific level is crucial to developing interventions aimed at improving earlier diagnosis.
